# Supplementary figures and images for: Impact of Leavening Agent and Wheat Variety on Bread Organoleptic and Nutritional Quality
Source: Microorganisms. 2022 Jul 14;10(7):1416. doi: 10.3390/microorganisms10071416 (PMC9317705; doi:10.3390/microorganisms10071416)

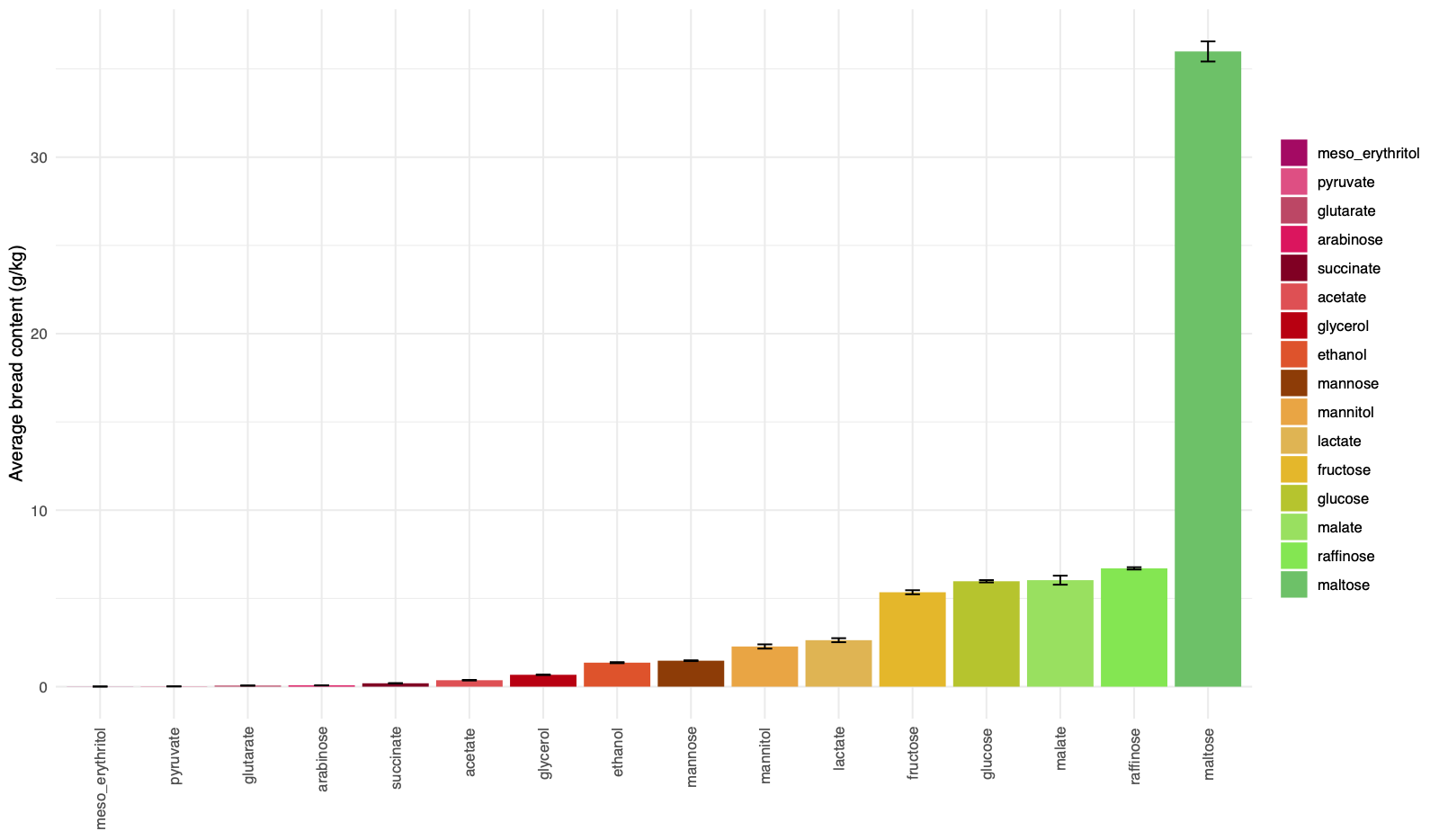

Supplement: Supplementary file 1 [file microorganisms-10-01416-s001.zip › microorganisms-1723725-supplementary/figureS1_carbohydrates_distribution_rd[1].png]

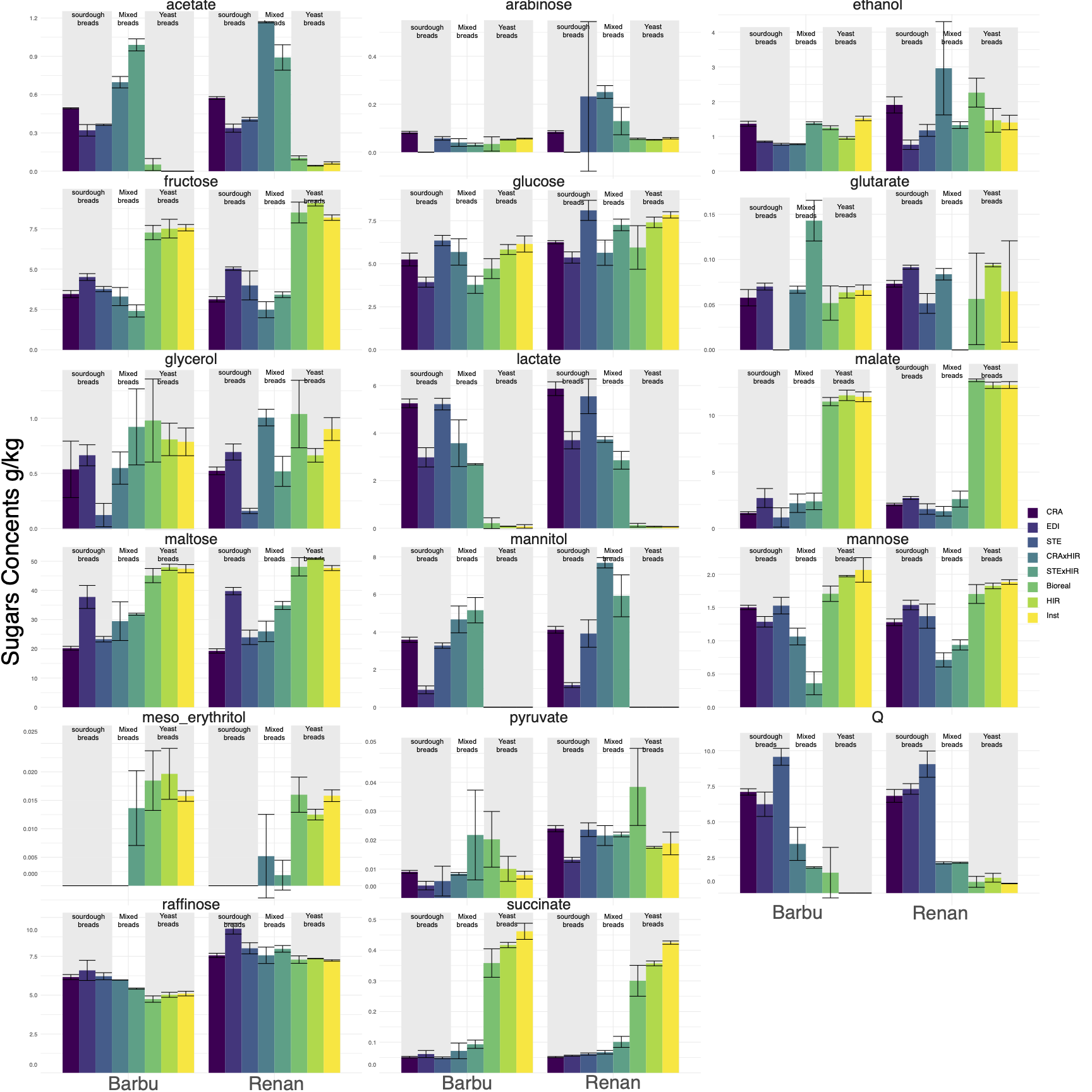

Supplement: Supplementary file 1 [file microorganisms-10-01416-s001.zip › microorganisms-1723725-supplementary/FigureS2_carbohydrates_detailed[1].png]

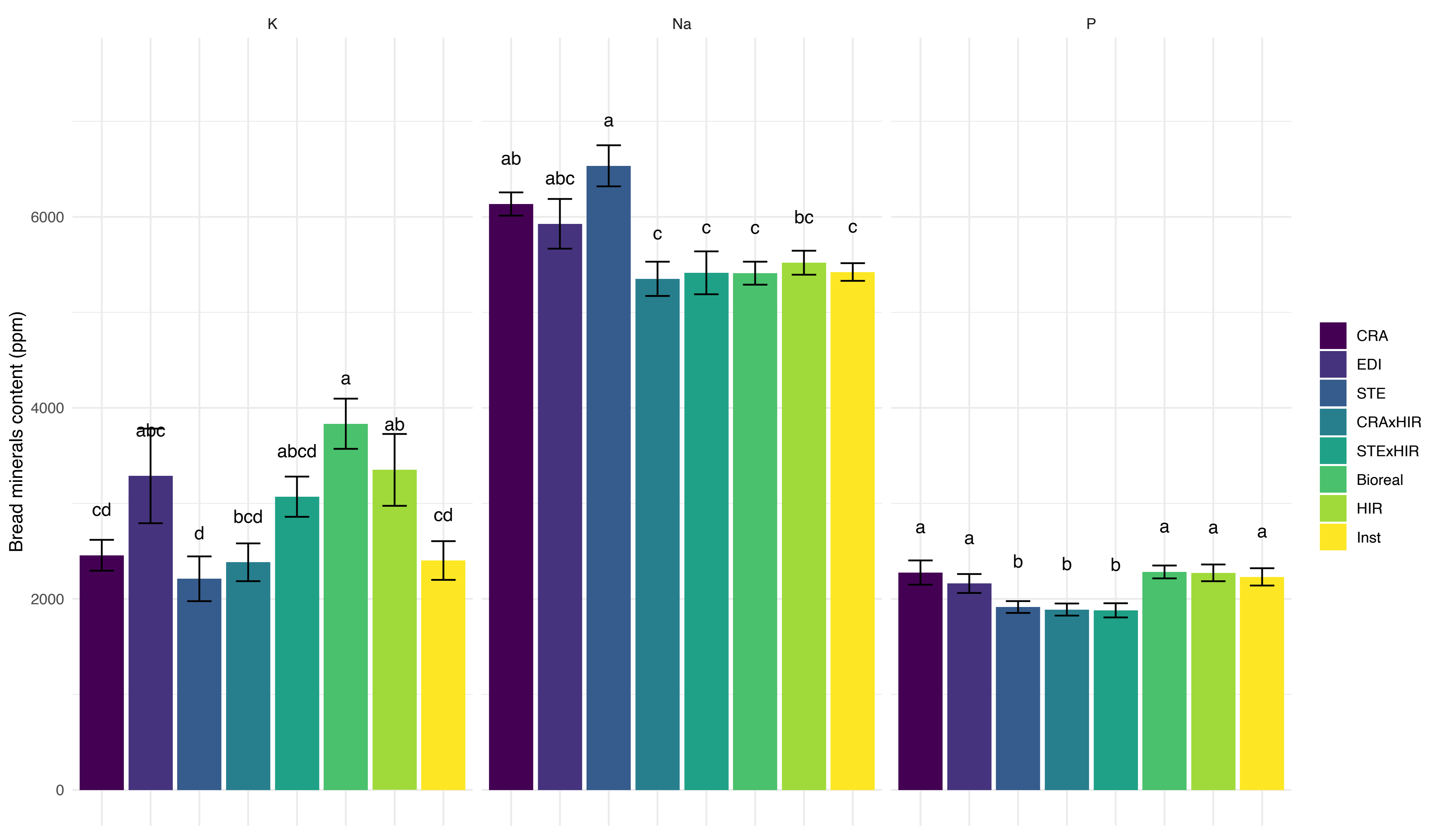

Supplement: Supplementary file 1 [file microorganisms-10-01416-s001.zip › microorganisms-1723725-supplementary/figureS3_main_minerals_rd[1].pdf]
